# Span-Morphing Wing Using Multistable Honeycomb Metamaterial Structures

**DOI:** 10.3390/ma19122678

**Published:** 2026-06-22

**Authors:** Ruixin Wang, Bin Niu

**Affiliations:** State Key Laboratory of High-performance Precision Manufacturing, School of Mechanical Engineering, Dalian University of Technology, Dalian 116024, China; wangruixin@mail.dlut.edu.cn

**Keywords:** multistable honeycomb structure, span-morphing wing, bistable curved beam, negative stiffness

## Abstract

**Highlights:**

- A span-morphing wing section based on multistable honeycomb structures is proposed.
- A nonlinear force–displacement model is established for multistable honeycomb structures with support stiffness considered.
- The wing achieves 35.6% span-morphing with validated bidirectional functionality.

**Abstract:**

Conventional span-morphing wings are often constrained by structural complexity, heavy weight, and discontinuous aerodynamic surface. Although flexible honeycomb and lattice structures offer lightweight solutions, they usually require external loads to maintain the deformed configuration and often exhibit limited stability under large deformation. In this study, a span-morphing wing section based on multistable honeycomb structures is proposed. The multistable honeycomb acts as the core deformation–load-bearing module, enabling multistage reversible spanwise reconfiguration through the bistable transition of cosine curved beams and the support of honeycomb structures. An equivalent nonlinear force–displacement model is derived to describe the structural response. Finite element analysis and fluid–structure interaction analysis are conducted to evaluate its mechanical and aerodynamic performance, while prototype fabrication and bidirectional morphing experiments are performed to demonstrate its functional feasibility. The results show that the proposed wing section achieves prescribed multistage state transitions, effectively regulates lift through span variation, and maintains good structural strength under typical aerodynamic loads. These findings demonstrate the potential of multistable honeycomb structures for lightweight and stable span-morphing wing design.

## 1. Introduction

Morphing aircraft can actively alter their aerodynamic shape or structural configuration to meet the requirements of different flight missions and operating conditions. They are therefore regarded as a promising direction for improving mission adaptability, aerodynamic efficiency, and environmental adaptability [1,2,3]. Among various morphing concepts, span-morphing wings are of particular interest because changes in wingspan directly affect wing area, aspect ratio, and induced drag. As a result, span variation offers clear benefits for low-speed lift enhancement, high-speed drag reduction, endurance improvement, and maneuverability enhancement [4,5,6]. This capability is especially attractive for small unmanned aerial vehicles and emerging green aircraft, for which efficient operation over a wide range of flight conditions is highly desirable [7,8,9].

Despite these advantages, the engineering realization of span-morphing wings remains challenging. Most existing designs rely on mechanical solutions such as telescopic beams, nested wing segments, sliding guide rails, and locking mechanisms [5,6]. Although these approaches can directly achieve span variation, they often require additional internal space, actuation systems, and transmission mechanisms, which increase structural mass and system complexity. A central challenge in the development of span-morphing wings is therefore to achieve large span variation while maintaining lightweight design, structural simplicity, and configuration stability.

In recent years, mechanical metamaterials have attracted increasing attention because their mechanical properties can be controlled by structural topology and geometric design [10,11,12,13,14,15,16,17,18]. Through unit-cell or microstructure design, these materials can achieve special and tunable responses, including a negative Poisson’s ratio [19,20], zero Poisson’s ratio [21], negative stiffness [22], anisotropic stiffness [23,24], and programmable deformation [25]. These properties make mechanical metamaterials promising for lightweight structures, adaptive structures, and shape-morphing systems. Among them, zero Poisson ratio (ZPR) and negative Poisson ratio (NPR) honeycomb and lattice structures have emerged as important candidates for morphing wings [26,27,28,29,30,31,32]. For example, NPR honeycomb structures have been used in chord- and camber-morphing wings [26,29], while ZPR honeycomb and lattice structures have been applied to span- and camber-morphing configurations [30,31,32].

However, flexible honeycomb and lattice structures still have important limitations. Because they rely mainly on elastic deformation, external loads are often required to maintain the deformed configuration after large shape changes. Their ability to lock and preserve a target shape is therefore limited. In addition, ZPR and NPR structures may lose their characteristic deformation behavior under large deformation [15,33]. Multistable structures provide a possible solution to this problem. Through suitable geometric or parametric design, they can possess multiple stable states and thus offer stable and reconfigurable deformation [34,35,36,37,38,39,40,41]. When multistable structures are combined with honeycomb structures, multistable honeycomb (MSHC) structures can be obtained [42,43,44,45,46]. These structures retain large reversible deformation capability while providing improved stiffness and configuration stability, making them promising candidates for integrated deformation–load-bearing applications in morphing wings. Nevertheless, their application to span-morphing wings remains limited. Most existing studies have focused on conceptual morphing capability, while design-oriented nonlinear modeling, aero-structural evaluation, and experimental validation at the wing-section level are still scarce. As a result, systematic exploration of how MSHC structures can be used for lightweight, stable, and load-bearing span-morphing is still lacking.

To address this gap, a span-morphing wing section based on MSHC structures is proposed in this study. Unlike ZPR/NPR honeycomb or compliant-lattice morphing concepts that mainly rely on continuous elastic deformation, the proposed design uses the stable-state transition of bistable curved beams to achieve discrete spanwise morphing states that can be maintained without continuous external loading. Compared with previous multistable metamaterial studies that mainly focused on configuration design and flexible morphing capability, this work integrates a multistable honeycomb structure into a wing-section-level span-morphing design, achieving functional integration of load-bearing and deformation. The study was organized as follows. First, an equivalent nonlinear force–displacement model considering finite support stiffness was derived to characterize the mechanical response of the MSHC structure and guide the prescribed sequential morphing design. Then, finite element analysis was performed to evaluate the sequential deformation behavior of the wing section. Subsequently, fluid–structure interaction analysis was conducted to investigate the aerodynamic response and load-bearing feasibility under typical aerodynamic loads. Finally, fabrication, assembly, and bidirectional morphing experiments were carried out to demonstrate the multistage reversible span-morphing capability and engineering feasibility of the proposed design. The proposed design provides a new approach for the development of lightweight and stable span-morphing wings.

## 2. Span-Morphing Wing Section Based on Multistable Honeycomb Structures

This section presents the design and mechanical modeling of the proposed span-morphing wing section. The structural concept is first introduced to clarify how the MSHC structure is integrated into the wing and how reversible span-morphing is achieved. The nonlinear force–displacement model is then developed for the MSHC structure, with support stiffness considered. This modeling step is essential because the state-transition behavior of the curved beams depends strongly on the boundary constraints provided by the surrounding honeycomb. The model therefore provides a mechanics-based basis for the subsequent sequential morphing design.

### 2.1. Conceptual Design of the Span-Morphing Wing Section

**Figure 1:**
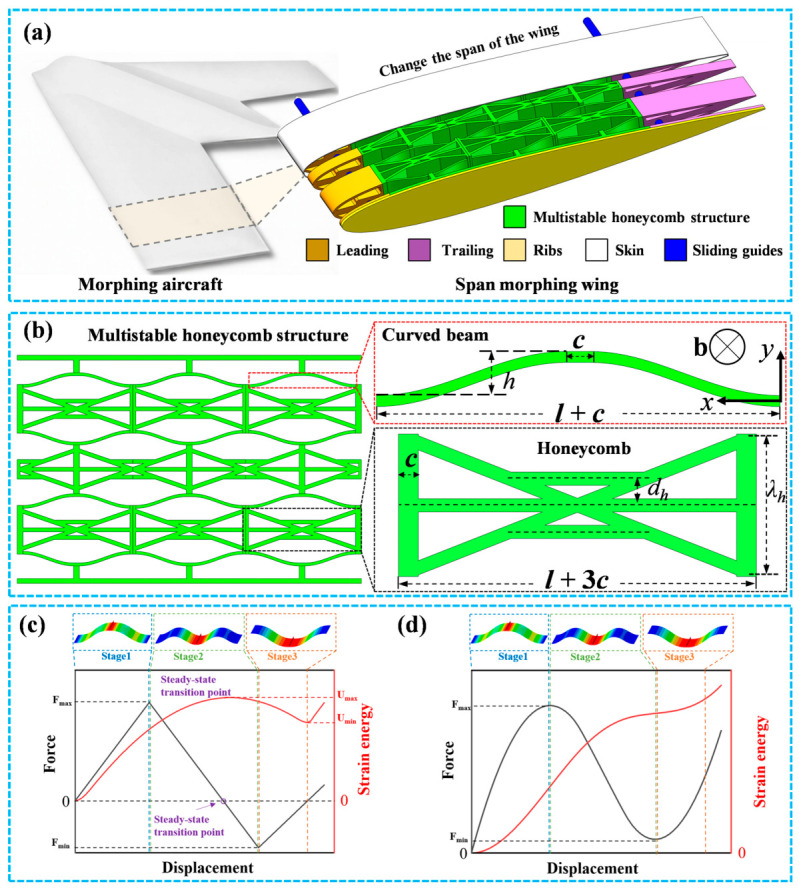
Conceptual model of the span-morphing wing. (**a**) Structural configuration of the span-morphing wing. (**b**) Structural configuration of the MSHC structure. (**c**) Schematic illustration of the force–displacement and strain-energy characteristics of a bistable cosine curved beam. (**d**) Schematic illustration of the force–displacement and strain-energy characteristics of a monostable cosine curved beam. (Reprinted with permission from Ref. [43]. Copyright 2025 Elsevier Ltd.)

The conceptual model of the proposed span-morphing wing section is shown in Figure 1a. A NACA0010 airfoil was adopted. To achieve reversible spanwise extension and contraction, MSHC structures were embedded within the wing as the core deformation–load-bearing modules, as indicated by the green region in Figure 1a. Leading-edge and trailing-edge structures were connected to the MSHC on the two chordwise sides, while two ribs were arranged at both spanwise ends to maintain boundary integrity. Sliding guides are shown as the blue components in Figure 1a. They were used to form sliding pairs with the leading-edge, trailing-edge, and rib components. The ends of the sliding guides were fixed to the lower rib. Therefore, the guides could support the wing section in the out-of-plane direction. During spanwise morphing, the sliding pairs allow relative motion along the span direction. At the same time, they limit out-of-plane relative motion. This design helps maintain geometric compatibility and improves the out-of-plane bending stiffness of the wing section.

Span-morphing is governed by the internal MSHC structure, in which the cosine curved beams serve as the bistable deformation elements and the surrounding honeycomb provides the boundary constraints required for state transition. To clarify the deformation mechanism, the monostable and bistable behaviors of the cosine curved beam are schematically illustrated in Figure 1c,d. Previous studies have shown that cosine curved beams can exhibit snap-through instability and negative-stiffness behavior when a force is applied near their middle region [37,43,45,47]. As shown in Figure 1c, a bistable curved beam has two stable equilibrium configurations. When the compression displacement exceeds the transition point, the beam undergoes a snap-through process and remains in the second stable state without continuous external loading. In contrast, as shown in Figure 1d, the monostable curved beam has only one stable equilibrium configuration and returns to its initial state after the external load is removed. For cosine curved beams, the structural parameter *Q* = *h*/*t* is an important factor governing this behavior, where *h* and *t* are the height and thickness of the curved beam structure, respectively. When *Q* > 2.31, the curved beam is a bistable structure; otherwise, it is a monostable self-restoring structure [37,43,45,47]. The stable states of the curved beams allow the deformed configuration to be maintained without continuous external loading, which is important for span-morphing wings that require both large reversible deformation and configuration stability.

### 2.2. Nonlinear Mechanical Behavior of the Multistable Honeycomb Structure

**Figure 2:**
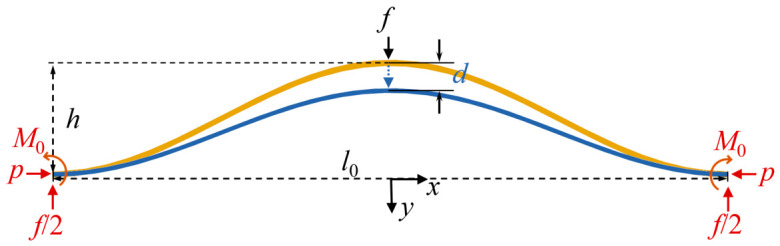
Free-body diagram of the curved beam in the MSHC structure. Adapted from Ref. [48].

To apply MSHC structures to the design of a span-morphing wing section, a nonlinear force–displacement model must first be established. In the MSHC structure considered in this study, the cosine curved beam is the primary component responsible for large deformation and snap-through transition. However, its two ends are connected to the surrounding honeycomb rather than to ideal rigid supports, as shown in Figure 1b. Therefore, the ideal fixed-end assumption cannot accurately describe the interaction between the curved beam and the supporting honeycomb. The Euler–Bernoulli beam theory was used to formulate this problem [47]. The free-body diagram of the curved beam in the MSHC structure is shown in Figure 2. Here, the positive *x*-axis is defined as pointing to the right, and the positive *y*-axis as pointing downwards. The initial shape of the curved beam structure is [47]:

(1) $$\bar{w}(x)=\frac{h}{2}\left[\cos\left(2\pi \frac{x}{l_{0}}\right)-1\right].$$

When an external force *f* is applied at the midpoint of the curved beam, an axial compressive load *p* is induced in the beam. Under the Euler-Bernoulli assumption, the beam is considered inextensible to the first-order approximation, and its governing equation can be written as:

(2) $$EI\frac{d^{2}w}{dx^{2}}+p\cdot d=M_{0}-p\cdot h-\frac{f\cdot x}{2},$$

where *d* is the displacement in the *y*-direction at the loading point, *E* is the Young’s modulus of the beam material, and *I* is the second moment of area of the beam cross-section.

**Figure 3:**
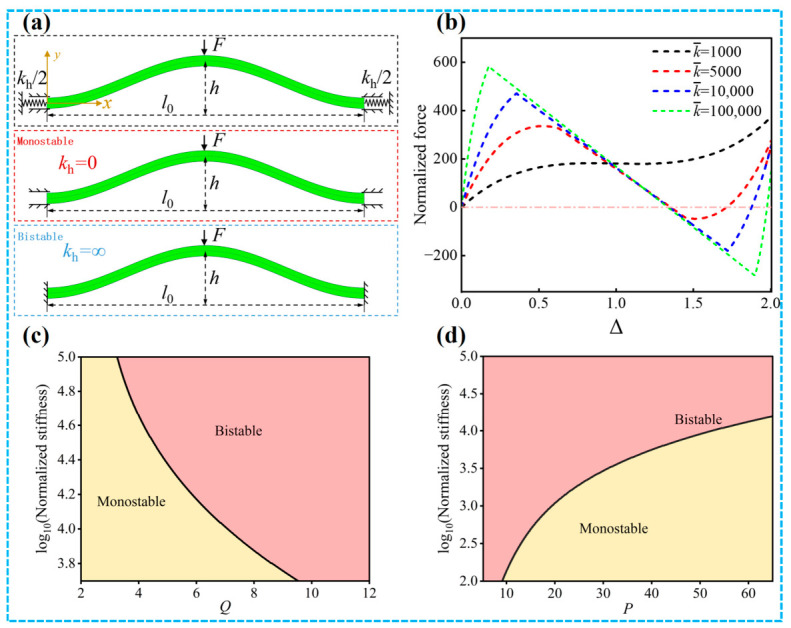
Nonlinear mechanical analysis of the curved beam in the MSHC structure. (**a**) Boundary support stiffness of the curved beam. (**b**) Force–displacement responses under different support stiffnesses. (**c**) Effects of *Q* and support stiffness on monostability and bistability. (**d**) Effects of *P* and support stiffness on monostability and bistability.

During snap-through deformation, the support compliance that most directly affects the bistability of the curved beam is the relative lateral displacement of its two ends. This displacement changes the projected distance between the two supports, thereby modifying the axial compression state and the force–displacement response of the beam. For this reason, the surrounding honeycomb was represented in the reduced analytical model by an equivalent lateral support stiffness. Based on this equivalent representation, the lateral compliance of the supporting honeycomb was modeled by two support springs, each with a stiffness of *k*_h_/2, as shown in Figure 3a. When *k*_h_ = 0, the lateral movement of the ends is unconstrained, and the curved beam tends to exhibit monostable behavior. When *k*_h_ = ∞, the ends become approximately fixed, and the curved beam exhibits a typical bistable response. Therefore, the mechanical response of the curved beam is governed by both its geometric parameters and the equivalent support stiffness. It should be noted that *k*_h_ is not an additional physical spring, but an equivalent stiffness used to represent the dominant in-plane compliance of the supporting honeycomb. Other deformation components of the support are neglected in this reduced analytical model for simplicity.

Based on the above simplification, the solution of Equation (2), and the following boundary conditions of the curved beam,

(3) $$w(0)=w(l)=0,{\left(\frac{dw}{dx}\right)}_{x=0}={\left(\frac{dw}{dx}\right)}_{x=l}=0\;,$$

the deflection of the deformed beam in the range of 0 ≤ *x* ≤ *l*_0_⁄2 can be obtained as follows [48]:

(4) $$\begin{array}{c} w=\bar{w}+d=\frac{h}{2}\frac{El{\left(\frac{2\pi }{l}\right)}^{2}}{El{\left(\frac{2\pi }{l}\right)}^{2}-p}\left[1-\cos\left(\frac{2\pi x}{l}\right)\right]\\ +\frac{\left[\cos\left(\frac{l}{2}\sqrt{\frac{p}{EI}}\right)-1\right]\left[\cos\left(x\sqrt{\frac{p}{EI}}\right)-1\right]f}{2p\sqrt{\frac{p}{EI}}\sin\left(\frac{l}{2}\sqrt{\frac{p}{EI}}\right)}\\ +\frac{F}{2p}\left[\sqrt{\frac{EI}{p}}\sin\left(x\sqrt{\frac{p}{EI}}\right)-x\right] \end{array}$$

where *l* denotes the current projected distance between the two end supports of the curved beam. When the curved beam is subjected to the midpoint load *f* (see Figure 2), relative horizontal displacement occurs at the beam ends. Therefore, *l* can be expressed as:

(5) $$l=\lambda l_{0}.$$

where *λ* is the normalized length ratio. According to the line integral formula, the length of the cosine curved beam structure can be expressed as [41,47]:

(6) $$s=\int_{0}^{l}\sqrt{1+{\left(\frac{dw}{dx}\right)}^{2}}dx\approx \int_{0}^{l}\left[1+\frac{1}{2}{\left(\frac{dw}{dx}\right)}^{2}\right]\;dx.$$

Based on the orthogonality of trigonometric functions, further simplification of Equation (5) yields [49]:

(7) $$s=l+\sum_{j=1}^{\infty }\frac{A_{j}^{2}N_{j}^{2}}{4}.$$

where *A_j_* is the dimensionless amplitude of the *j*-th buckling mode and represents the contribution of this mode to the curved-beam deformation, while *N_j_* is the corresponding dimensionless eigenvalue that determines the spatial form of the *j*-th mode. Due to the presence of the lateral springs, a coupled relationship is established between the change in the distance between the end supports of the curved beam and the axial force:

(8) $$p=Ebt\left(1-\frac{s}{s_{0}}\right)=k_{h}\left(l-l_{0}\right).$$

where *E* is the elastic modulus of the curved beam structure, and *b* and *t* are the cross-sectional width and thickness of the curved beam structure, respectively.

A displacement-based variational method was then used to calculate the force–displacement response of the curved beam. First, the following variables are normalized:

(9) $$F=\frac{fl_{0}^{3}}{EIh},\;\Delta =\frac{d}{h},\;\bar{p}=\frac{pl_{0}^{2}}{EI},\;\bar{k}=\frac{k_{h}l_{0}^{2}}{EI},{\;U}_{b}=\frac{u_{b}l_{0}^{3}}{EIh^{2}},{\;U}_{s}=\frac{u_{s}l_{0}^{3}}{EIh^{2}},{\;U}_{f}=\frac{u_{f}l_{0}^{3}}{EIh^{2}},{\;U}_{k}=\frac{u_{k}l_{0}^{3}}{EIh^{2}}$$

where *I* is the second moment of inertia of the curved beam; *u_b_*, *u_s_*, *u_f_* and *u_k_* are the bending energy, axial compression energy, external-load energy and spring energy of the curved beam structure, respectively; and Δ is the dimensionless displacement in the y-direction at the loading point. In addition, let *x* ∈ [0, *l*] denote the coordinate along the line connecting the two supports in the current configuration, as shown in Figure 3a. The dimensionless coordinate is defined as:

(10) $$X=\frac{x}{l}.$$

Accordingly, the normalized arc length of the curved-beam structure with boundary support can be expressed as:

(11) $$\bar{s}=\frac{sl_{0}}{h^{2}}=\lambda \frac{l_{0}^{2}}{h^{2}}+\frac{1}{4\lambda }\sum_{j=1}^{\infty }(A_{j}N_{j}{)}^{2}.$$

From Equations (8) and (9), the arc-length change in the curved beam structure and its variation can be further derived as:

(12) $${\bar{s}}_{0}-\bar{s}=\frac{\bar{p}{\bar{s}}_{0}t^{2}}{12l_{0}^{2}},$$

(13) $$\delta s=-\frac{s_{0}k_{h}}{Ebt}\delta l.$$

To simplify the derivation, an intermediate variable is introduced [48]:

(14) $$\bar{M}=\sum_{j=1}^{\infty }(A_{j}N_{j}{)}^{2},$$

Here, $\bar{M}$ represents the modal contribution to the normalized arc length of the deformed curved beam. It collects the squared contributions of all buckling modes and is introduced to simplify the arc-length constraint. Substituting Equation (14) into the normalized arc-length expression in Equation (11) and using the arc-length constraint in Equation (12), $\bar{M}$ can be expressed as:

(15) $$\bar{M}=4\lambda \left({\bar{s}}_{0}-\lambda \frac{l_{0}^{2}}{h^{2}}-\frac{\bar{p}{\bar{s}}_{0}t^{2}}{12l_{0}^{2}}\right).$$

Another intermediate variable is introduced:

(16) $$\bar{C}=\frac{l_{0}^{2}}{h^{2}}-\frac{\bar{M}}{4\lambda^{2}},$$

By taking the variation of Equation (11), substituting the variation of $\bar{M}$ and combining it with the spring-induced arc-length variation in Equation (13), the following expression can be obtained [47,48]:

(17) $$\left(\bar{C}+\frac{{\bar{s}}_{0}\bar{k}t^{2}}{12l_{0}^{2}}\right)\delta \lambda =-\frac{\sum_{j=1}^{\infty }{N_{j}}^{2}A_{j}\delta A_{j}}{2\lambda }.$$

A further intermediate variable is introduced:

(18) $$\bar{R}=\bar{C}+\frac{{\bar{s}}_{0}\bar{k}t^{2}}{12l_{0}^{2}}.$$

Accordingly, the variational relationship between the dimensionless arc-length parameter *λ* and the modal coefficients in Equation (17) can be written as:

(19) $$\delta \lambda =-\frac{\sum_{j=1}^{\infty }{N_{j}}^{2}A_{j}\delta A_{j}}{2\lambda \bar{R}}.$$

With the support stiffness included, the total potential energy consists of the bending energy *u_b_*, axial compression energy *u_s_*, spring energy *u_k_*, and external-load energy *u_f_*. These energy terms can be expressed as:

(20) $$u_{b}=\frac{EI}{2}\int_{0}^{l_{0}}{\left(\frac{d^{2}w}{dx^{2}}-\frac{d^{2}w_{0}}{dx^{2}}\right)}^{2}dx,$$

(21) $$u_{f}=-\int_{0}^{d}f(\Delta )\cdot d\Delta ,$$

(22) $$u_{k}=\frac{1}{2}k_{h}{\left(l-l_{0}\right)}^{2}.$$

The variation in the compressive energy in the beam is given by

(23) $$\partial (u_{s})=-p\partial (s)=\frac{ps_{0}k_{h}}{Ebt}\partial l.$$

The corresponding normalization variational forms are given by Equations (24)–(29).

(24) $$\delta \Pi =\delta \left(U_{b}+U_{s}+U_{k}+U_{f}\right)=0.$$

(25) $$\delta (U_{b})=\delta \left[\frac{1}{2}\int_{0}^{l}{\left(\frac{d^{2}\bar{W}}{dx^{2}}-\frac{d^{2}W}{dx^{2}}\right)}^{2}dx\right],\;\delta (U_{s})=-N^{2}\delta (S),\;\delta (U_{f})=-F\delta (\Delta ).$$

(26) $$\delta U_{b}\approx \frac{N_{1}^{4}}{2\lambda^{2}}\left(\frac{A_{1}}{\lambda^{2}}-A_{1}^{\left(0\right)}\right)\delta A_{1}+\sum_{j=2}^{\infty }\frac{N_{j}^{4}}{2\lambda^{4}}A_{j}\delta A_{j}.$$

(27) $$\delta U_{s}=-\frac{\bar{p}{\bar{s}}_{0}\bar{k}t^{2}}{24l_{0}^{2}\lambda \bar{R}}\sum_{j=1}^{\infty }N_{j}^{2}A_{j}\delta A_{j}.$$

(28) $$\delta U_{f}=2F\underset{j=1,5,9,\ldots }{\sum }\delta A_{j}.$$

(29) $$\delta U_{k}=\frac{l_{0}^{2}(1-\lambda )\bar{k}}{2h^{2}\lambda \bar{R}}\sum_{j=1}^{\infty }N_{j}^{2}A_{j}\delta A_{j}.$$

By combining the above terms and introducing the intermediate variables in Equation (30), the variation in the total potential energy can finally be expressed as Equation (31).

(30) $$G_{j}=\frac{N_{j}^{4}}{2\lambda^{4}}-\frac{\bar{p}s_{0}\bar{k}t^{2}N_{j}^{2}}{24l_{0}^{2}\lambda \bar{R}}+\frac{l_{0}^{2}(1-\lambda )\bar{k}N_{j}^{2}}{2h^{2}\lambda \bar{R}}.$$

(31) $$\delta \Pi =\left(G_{1}A_{1}-\frac{N_{1}^{4}A_{1}^{\left(0\right)}}{2\lambda^{2}}+2F\right)\delta A_{1}+\underset{j=5,9,\ldots }{\sum }\left(G_{j}A_{j}+2F\right)\delta A_{j}\;+\underset{j=2,3,4,6,7,\ldots }{\sum }G_{j}A_{j}\delta A_{j}=0$$

The equilibrium equation corresponding to Equation (31) can be solved in three cases [47,48]. In Case 1, only the symmetric modes with *j* = 1,5,9 … are retained. In Case 2, asymmetric deformation is allowed through the participation of the second mode. In Case 3, the second mode is constrained, while the third symmetric mode is allowed to appear. Since the second mode generally does not lead to a stable bistable response, only Cases 1 and 3 are considered in this study. For Case 1, Equation (32) is obtained from Equation (31), and a quadratic equation for the external load *F* is derived as Equation (33). For Case 3, Equation (34) is obtained by imposing *A*_3_ = 0. Substituting Equation (34) into Equation (33) gives the external-load expression in Equation (35).

(32) $$A_{1}=\frac{N_{1}^{4}A_{1}^{\left(0\right)}}{2\lambda^{2}G_{1}}-\frac{2F}{G_{1}},\;{\;A}_{j}=-\frac{2F}{G_{j}},\;\;j=5,9,\ldots$$

(33) $$\left(\underset{j=1,5,9,\ldots }{\sum }\frac{4N_{j}^{2}}{G_{j}^{2}}\right)F^{2}-\frac{2N_{1}^{6}A_{1}^{\left(0\right)}}{\lambda^{2}G_{1}^{2}}F+\frac{N_{1}^{10}(A_{1}^{\left(0\right)}{)}^{2}}{4\lambda^{4}G_{1}^{2}}-\bar{M}=0.$$

(34) $$\bar{p}=\frac{24l_{0}^{2}\bar{R}}{{\bar{s}}_{0}\bar{k}t^{2}}\left[\frac{l_{0}^{2}(1-\lambda )\bar{k}}{2h^{2}\bar{R}}+\frac{N_{3}^{2}}{2\lambda^{3}}\right].$$

(35) $$\left(\underset{j=1,5,9,\ldots }{\sum }\frac{4N_{j}^{2}}{G_{j}^{2}}\right)F^{2}-\frac{N_{1}^{6}}{\lambda^{2}G_{1}^{2}}F+\frac{N_{1}^{10}}{16\lambda^{4}G_{1}^{2}}+N_{3}^{2}A_{3}^{2}-\bar{M}=0.$$

Based on the above derivation, the mechanical response of the curved beam under different boundary support conditions can be obtained. Figure 3b shows the dimensionless force–displacement responses for different support stiffnesses, calculated with *h* = 8 mm, *L* = 64 mm, and *t* = 0.8 mm. The results indicate that support stiffness strongly affects bistable behavior. As the support stiffness decreases, a curved beam with bistable potential gradually transitions to monostable behavior.

To further examine the bistability boundary, a parametric analysis was performed in terms of the dimensionless stiffness $\bar{k}$ and the geometric parameters *P* and *Q*, where *P* = *L*/*t* and *Q* = *h*/*t*. First, $\bar{k}$ was varied from 100 to 100,000 and *Q* from 2 to 12, while *t* = 0.8 mm and *l* = 120 mm were fixed so that *P* remained constant. Then, $\bar{k}$ was varied from 5000 to 100,000 and *P* from 5 to 65, while *t* = 0.8 mm and *h* = 4 mm were fixed so that *Q* remained constant. The results are shown in Figure 3c,d. As $\bar{k}$ increases, the minimum *Q* required for bistability decreases. When the support approaches the ideal fixed condition, the monostability–bistability boundary is a straight line at *Q* = 2.31 [47]; with finite support stiffness, it becomes a nonlinear curve. Figure 3d further shows that *P* also significantly affects the bistability boundary. Therefore, *P* and *Q* can be used as key dimensionless parameters to characterize the bistable behavior of the cosine curved beam.

When the MSHC structure consists of multiple cells, adjacent cells can be approximated by symmetric boundary conditions. The honeycomb walls therefore mainly carry axial tensile and compressive loads, and the supporting honeycomb connected to the curved beam can be equivalently modeled as an axial tension–compression spring with stiffness given by Equation (36), where *E*_h_ and *c* denote the elastic modulus and wall thickness of the supporting honeycomb, respectively. By substituting this stiffness into Equation (9), the dimensionless force–displacement relationship of a single curved beam under support stiffness *k_h_* can be obtained and then converted into dimensional form.

(36) $$k_{h}=\frac{E_{h}A}{c}.$$

**Figure 4:**
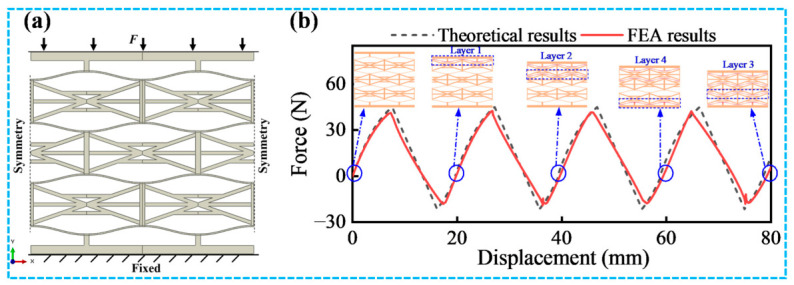
Comparison between theoretical and finite element results for the nonlinear mechanical response of the MSHC structure. (**a**) Finite element analysis setup of the 2 × 2 MSHC structure. (**b**) Comparison between theoretical and finite element results.

To validate the theoretical model, the 2 × 2 MSHC structure shown in Figure 4a was analyzed and compared with finite element results. The finite element simulation was conducted in Abaqus using a “Dynamic, Implicit” analysis step with geometric nonlinearity enabled through the NLGEOM option. A displacement-controlled loading method was adopted. The bottom surface of the model was fixed, symmetry boundary conditions were applied to the left and right side surfaces, and a downward displacement was prescribed on the top surface at a loading rate of 5 mm/s. The reaction force corresponding to the imposed displacement was extracted to obtain the force–displacement response. The model was meshed using 8-node linear brick elements with reduced integration (C3D8R). A mesh convergence analysis was performed to ensure numerical accuracy. The final mesh size was 1.5 mm for the supporting honeycomb and was refined to 0.4 mm for the curved-beam regions, where large deformation and stress concentration occur during snap-through. Polylactic Acid (PLA) and thermoplastic polyurethane (TPU) were selected as the materials for the supporting honeycomb and curved beam, respectively. PLA was assigned a Young’s modulus of 2100 MPa, a Poisson’s ratio of 0.3, and a density of 1430 kg/m^3^. TPU was modeled in the linear elastic range, with a Young’s modulus of 58 MPa, a Poisson’s ratio of 0.45, and a density of 1200 kg/m^3^ [45].

Because this structure is a multilayer multistable system connected in series, its overall response cannot be obtained by direct superposition of the responses of individual curved-beam layers. A series-coupled nonlinear stiffness model was therefore adopted [43]. The equivalent nonlinear stiffness is given by Equation (37), where *m*_I_, *m*_II_, and *m*_III_ denote the numbers of unit layers in Stage I, Stage II, and Stage III, respectively. Here, Stages I, II, and III correspond to the initial positive-stiffness segment, the negative-stiffness segment, and the subsequent positive-stiffness segment in the bistable deformation process, as shown in Figure 3b. The equivalent displacement is given by Equation (38), where *δ*_eI_, *δ*_eII_, and *δ*_eIII_ denote the number of layers in the three stages. Following previous studies, the series-coupled nonlinear stiffness model assumes that only one unit layer enters Stage II, namely the negative-stiffness transition stage, at a given time [42,43,50]. This assumption allows the multilayer MSHC structure to be described through a sequential snap-through path. The overall force–displacement relationship is then obtained from Equation (39).

(37) $$K_{e}^{*}=\left\{\begin{array}{c} {\left(\frac{n\cdot m_{I}}{K_{I}}+\frac{n\cdot m_{\mathrm{III}}}{K_{\mathrm{III}}}\right)}^{-1},\;\;\;\;when\;m_{\mathrm{II}}=0\;,\;m_{I}+m_{\mathrm{III}}=m\\ {\left(\frac{-n\cdot m_{I}}{K_{I}}+\frac{n\cdot m_{\mathrm{II}}}{K_{\mathrm{II}}}+\frac{-n\cdot m_{\mathrm{III}}}{K_{\mathrm{III}}}\right)}^{-1},\;\;\;\;when\;m_{\mathrm{II}}=1\;,\;m_{I}+m_{\mathrm{II}}+m_{\mathrm{III}}=m \end{array}\right.$$

(38) $$\delta_{e}=m_{I}\delta_{\mathrm{eI}}+m_{\mathrm{II}}\delta_{\mathrm{eII}}+m_{\mathrm{III}}\delta_{\mathrm{eIII}},\;m_{\mathrm{II}}\leq 1\;,\;m_{I}+m_{\mathrm{II}}+m_{\mathrm{III}}=m$$

(39) $$F_{e}=K_{e}^{*}\delta_{e}$$

Figure 4b shows good agreement between the theoretical and finite element results in the peak-force positions, state-transition plateaus, and overall response trend. This confirms that the proposed analytical model can effectively describe the nonlinear mechanical behavior of the MSHC structure under finite boundary support conditions and provides a reliable basis for subsequent wing-section design.

## 3. Numerical Evaluation of the Wing Section

This section presents the numerical evaluation of the proposed wing section. First, the wing section is designed to achieve a prescribed morphing sequence through graded curved-beam thicknesses, and its sequential deformation behavior is analyzed. Then, one-way fluid–structure interaction analysis is conducted to evaluate the aerodynamic response, load-bearing capability, and structural deformation under typical aerodynamic loads. Together, these results provide a numerical assessment of the feasibility and performance of the proposed design.

### 3.1. Wing Section Design for a Prescribed Morphing Sequence

To verify the structural feasibility and sequential deformation capability of the proposed span-morphing wing section, a finite element model was established. The wing section had a chord length of 577 mm, an initial span of 270 mm, and four layers of bistable units, corresponding to a total span stroke of 96 mm. The material parameters of TPU and PLA were the same as those described in Section 2.2. In the wing-section model, TPU was assigned to the curved-beam units to provide the required compliance for snap-through deformation, whereas PLA was assigned to the leading edge, trailing edge, ribs, and auxiliary supporting components to maintain the structural stiffness of the non-morphing regions. This material arrangement preserved compliance in the morphing region while maintaining the basic load-carrying stiffness of the overall structure.

**Figure 5:**
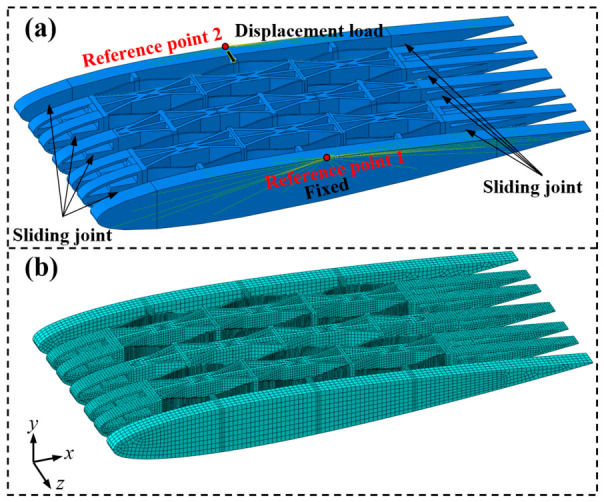
Simplified finite element model setup of the span-morphing wing section. (**a**) Simplified geometric model and boundary conditions, where the yellow lines indicate the coupling constraints used to connect the reference points with the corresponding rib components. (**b**) Finite element mesh of the wing-section model.

The simplified finite element model and mesh of the span-morphing wing section are shown in Figure 5. As shown in Figure 5a, the finite element model retained the main structural components, while the guide rails were removed for simplification. To reproduce the connection with the rigid guide rails, sliding constraints in the *z*-direction were applied to the internal regions of the leading-edge and trailing-edge components. The lower and upper ribs are connected to Reference points 1 and 2 using “coupling” constraints, respectively. Reference point 1 was fixed, while a prescribed displacement in the *z*-direction was applied to reference point 2. The loading speed was set to 1 mm/s until the maximum stroke of 96 mm was reached, and was then changed to −1 mm/s until the wing section returned to its initial configuration. The analysis step was consistent with that described in Section 2.2. The model was meshed using 8-node linear brick elements with reduced integration (C3D8R). As shown in Figure 5b, the mesh size of the curved-beam regions was refined to 0.4 mm to accurately capture the snap-through deformation, while a mesh size of 3 mm was used for the remaining structural components to improve computational efficiency.

**Figure 6:**
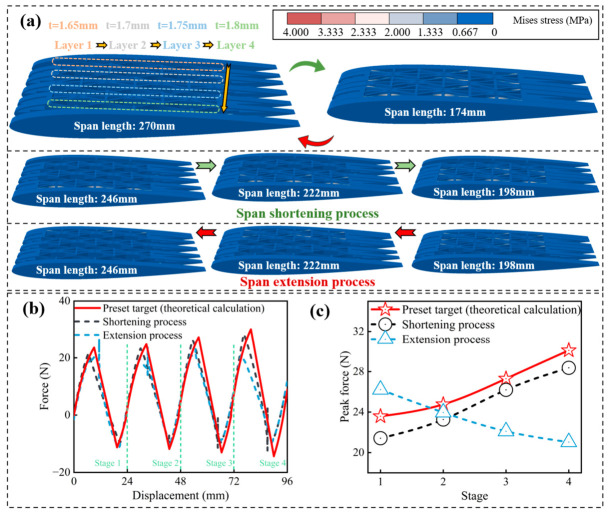
Analysis of the deformation process of the span-morphing wing. (**a**) Stress contours. (**b**) Force–displacement curves. (**c**) Peak forces at different stages of deformation.

To realize multistage state transitions in a prescribed order during spanwise shortening and extension, the four layers of multistable honeycomb units were assigned cosine curved beams with thicknesses of 1.65, 1.70, 1.75, and 1.8 mm, respectively, as shown in Figure 6a. The beam height and span were 12 and 104 mm. The graded thickness design was introduced to generate different transition thresholds among the four unit layers. A thicker curved beam generally requires a higher snap-through peak force, while a thinner beam tends to switch earlier under the same loading path. Therefore, the transition order of the unit layers can be programmed by assigning different beam thicknesses along the spanwise direction. As a result, the unit layers underwent successive state transitions under external loading, enabling controllable sequential deformation. Figure 6a shows four discrete state transitions during both shortening and extension. The detailed morphing process obtained from the corresponding finite element simulation is provided as Video S1 in the Supplementary Material. The resulting span stroke was 96 mm, corresponding to 35.6% of the maximum span, and the deformation sequence agreed with the design target. These results show that graded beam thickness can regulate the transition order and achieve ordered spanwise reconfiguration.

Figure 6b,c show the force–displacement response of the wing section during a complete shortening-extension cycle and the corresponding peak-force variation. The red curves represent the prescribed target calculated from the nonlinear mechanical model in Section 2.2. Good agreement is observed in both the deformation sequence and the mechanical response. All eight state transitions show clear periodicity, and the peak force changes progressively with the structural hierarchy. These results confirm that the graded MSHC structure enables multistage reversible span-morphing with a stable transition pattern, providing a structural basis for the subsequent aerodynamic analysis and prototype experiments.

### 3.2. Fluid–Structure Interaction Analysis of the Wing Section

**Figure 7:**
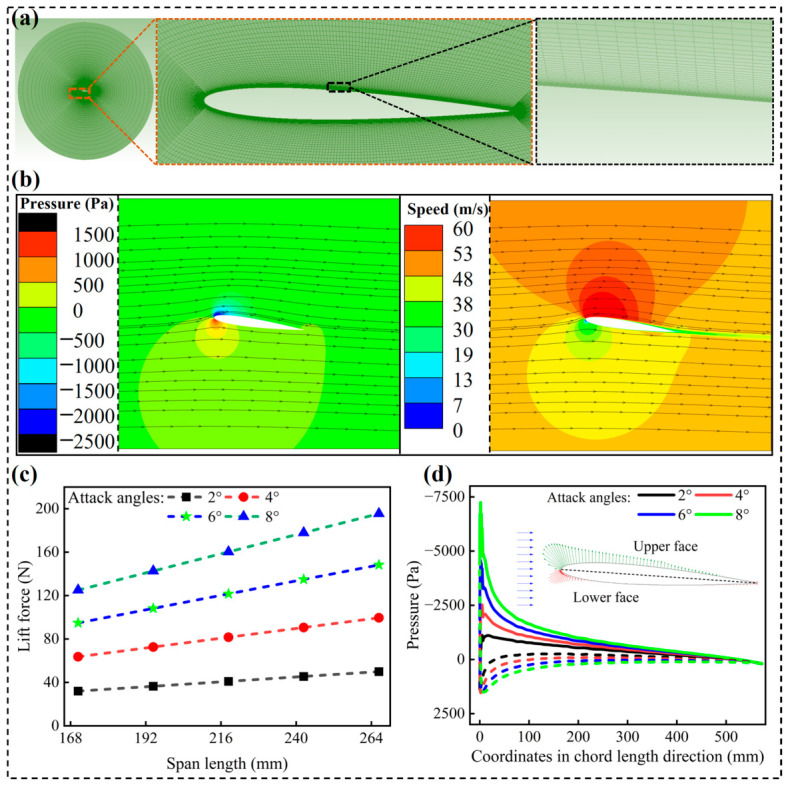
Flow-field analysis setup and results. (**a**) Computational domain and mesh. (**b**) Pressure and velocity distributions at an angle of attack of 8°. (**c**) Lift variation in the span-morphing wing. (**d**) Pressure distributions over the wing surface at different angles of attack.

To evaluate the aerodynamic performance and load-bearing feasibility of the proposed span-morphing wing section, a one-way fluid–structure interaction analysis was conducted with reference to the papers of Wu et al. [51], Zhang et al. [52] and Thangeswaran et al. [53]. The flow field was established in Fluent, as shown in Figure 7a. A cylindrical external domain was adopted, with a cross-sectional diameter of 15 chord lengths (8.65 m) and a spanwise length equal to the maximum span of the wing section (266.4 mm). The inlet velocity was set to 50 m/s, and a zero-pressure-gradient condition was applied at the outlet. The wing surface was defined as a no-slip wall, and the far-field boundary was set sufficiently far from the wing to reduce boundary interference. The angle of attack was prescribed by changing the inflow direction relative to the wing section. An O-type structured mesh with approximately 1.79 million elements was used. The minimum boundary-layer mesh height was 0.007 mm, with a growth rate of 1.2, corresponding to a *Y*+ value of about 1. A total of 30 layers were generated in the near-wall boundary layer to resolve the viscous sublayer. The inlet turbulence intensity was set to 5%, and the turbulence length scale was determined according to the characteristic chord length of the wing section. The ideal-gas assumption and the SST *k*-*ω* turbulence model were adopted to improve near-wall prediction accuracy. The simulations were conducted as steady-state Reynolds-averaged Navier–Stokes calculations using a pressure-based solver. The pressure–velocity coupling was solved using the SIMPLE algorithm, and second-order discretization schemes were used for the momentum and turbulence equations. The residual convergence criterion was set to 10^−5^, and the lift force was also monitored to ensure that the aerodynamic force reached a stable value. In addition, a mesh-independence analysis was carried out by comparing the lift results obtained from different mesh densities, and the selected mesh was found to provide a good balance between accuracy and computational cost.

Numerical analyses were then performed at angles of attack of 2°, 4°, 6°, and 8° for different span states. Figure 7b shows the pressure and velocity distributions at 8°, where a high-pressure region appears near the leading edge, a clear acceleration region develops over the upper surface, and a low-velocity wake forms behind the wing. Figure 7c shows that lift increases with angle of attack and varies significantly among different spanwise lengths at the same angle of attack. This confirms that the proposed MSHC-based span-morphing design can effectively regulate lift by changing the span. Figure 7d further shows the typical pressure distribution of negative pressure on the upper surface and positive pressure on the lower surface, with the pressure difference increasing as the angle of attack increases.

**Figure 8:**
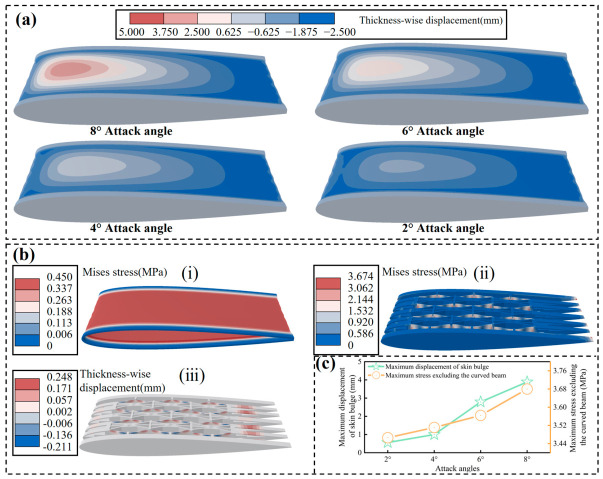
Finite element results under aerodynamic loading. (**a**) Thickness-direction displacement contours of the flexible skin at different angles of attack. (**b**) Results at an angle of attack of 8°: (i) stress distribution in the flexible skin, (ii) stress distribution in the wing structure, and (iii) thickness-direction displacement contours of the wing. (**c**) Maximum bulging displacement of the skin and maximum stress in the load-bearing structure at different angles of attack.

The aerodynamic pressure obtained from the flow-field analysis was then applied to the Abaqus finite element model to evaluate the structural response under typical flight loads. Based on the pressure distributions shown in Figure 7d, pressure point-cloud data were extracted at equally spaced locations from the upper and lower surfaces of the wing. These pressure data were then introduced into Abaqus through the Analytical Field function as spatially varying pressure fields and applied to the corresponding upper and lower wing surfaces. This procedure allowed the aerodynamic pressure calculated in Fluent to be used as the external load for the nonlinear structural analysis in Abaqus. A dynamic, implicit step was adopted, and the structure was meshed with C3D8R elements. The interaction of surface-to-surface contact includes both tangential and normal behaviors. The friction coefficient for tangential behavior is 0.45, which is within the reported range for TPU and PLA-based printed polymers [54,55]. The normal behavior was characterized as “Hard” contact. The fully shortened wing section was taken as the initial configuration and covered with a 4 mm flexible skin. A tensile load was then applied to drive the section from the fully shortened to the fully extended state. The skin was modeled as silicone rubber with a Shore hardness of 20 using a third-order Yeoh model (*C*_10_ = 0.10620, *C*_20_ = −0.015063, *C*_30_ = 0.0029714) [56], and was meshed with C3D8RH elements. Figure 8a shows that the skin becomes thinner during spanwise stretching and develops local bulging on the upper surface under aerodynamic suction, with bulging increasing with angle of attack. Under the most severe condition of 8°, Figure 8b shows that the maximum skin stress is 0.450 MPa and is distributed relatively uniformly, while the maximum wing stress is 3.674 MPa and is concentrated mainly in the curved-beam regions and at the trailing-edge tip. Figure 8c shows that both the maximum bulging displacement and the maximum stress in the main load-bearing structure increase with the angle of attack. The bulging displacement increases from 0.562 mm at 2° to 3.887 mm at 8°, corresponding to 0.099% and 0.682% of the chord length, respectively, while the maximum structural stress remains far below the material yield strength. These results indicate that the proposed wing section maintains sufficient strength and good shape-retention capability under typical aerodynamic loads, thereby satisfying the intended integrated deformation–load-bearing requirement.

It should be noted that the present analysis adopted a one-way fluid–structure interaction strategy, in which the aerodynamic pressure obtained from Fluent was mapped to the structural model in Abaqus. This treatment neglects the feedback of structural deformation on the aerodynamic field. However, the aerodynamic-load-induced deformation of the wing surface was relatively small in the present study. Under the most severe condition considered, the maximum bulging displacement of the flexible skin was only 3.887 mm, corresponding to 0.682% of the chord length. Therefore, the change in aerodynamic shape caused by the pressure load was limited relative to the characteristic chord length. For this reason, the one-way fluid–structure interaction approach was considered acceptable for the present feasibility-level evaluation of aerodynamic loading, structural deformation, and load-bearing capability. Nevertheless, two-way fluid–structure interaction analysis would be necessary for more accurate aeroelastic prediction when larger skin deformation, unsteady aerodynamic effects, or dynamic flight conditions are considered.

## 4. Fabrication, Assembly, and Functional Demonstration of Bidirectional Morphing

**Figure 9:**
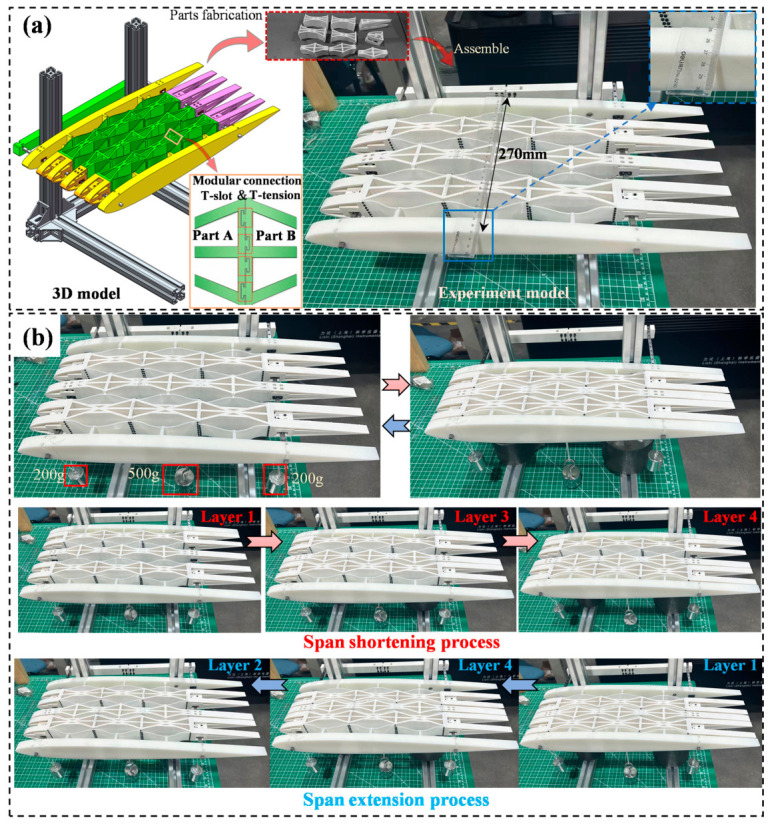
Fabrication and bidirectional deformation experiments of the span-morphing wing section. (**a**) Fabrication and assembly process. (**b**) Bidirectional deformation process under loading.

To further verify the engineering feasibility of the proposed design, fabrication, assembly, and bidirectional morphing experiments were carried out on the span-morphing wing section. First, the specific geometric dimensions of each component were determined from the three-dimensional model shown in Figure 9a. Then, modular T-slot and T-tenon interfaces arranged in staggered pairs were designed at the connection locations of the components to facilitate rapid assembly and disassembly. Finally, all functional modules were fabricated by additive manufacturing and assembled into the complete wing section. These experiments were intended to demonstrate the functional feasibility of the proposed concept rather than to exactly reproduce the transition sequence obtained in the numerical model. The main focus was placed on whether the assembled wing section could undergo reversible spanwise shortening and extension while retaining discrete state-transition behavior.

It should be noted that the nozzle diameter of the printer was 0.4 mm, which imposed certain limitations on the manufacturing accuracy of the graded-thickness curved beams. Therefore, the MSHC structure in the experimental specimen did not adopt the graded-thickness curved beams used in the simulation. Instead, cosine curved beams with a uniform thickness of 1.2 mm were used, and the material was TPU. The remaining supporting and connecting components were fabricated and assembled according to the design requirements. This treatment preserved the basic spanwise deformation mechanism of the MSHC structure while maintaining manufacturing feasibility.

After assembly, bidirectional deformation experiments were conducted to validate the functionality of the wing section. The experiments focused on the shortening and extension processes under external loading, as well as the state-transition capability of the MSHC structure in the assembled configuration. Figure 9b shows the bidirectional deformation process of the wing section under an external load of 0.9 kg. The fabricated specimen was able to undergo spanwise shortening and extension under external loads, indicating that the MSHC structure retained the intended deformation function in the span-morphing wing section. Although the thicknesses of the curved beams in all four layers were the same, 1.2 mm, different assembly errors in different layers resulted in different boundary conditions. This changed the peak force required for state transition in each curved-beam layer and led to eight discrete deformation events. During the span-shortening process, the state transitions occurred in the order of Layer 1, Layer 3, Layer 4, and Layer 2. During the span-extension process, the order changed to Layer 1, Layer 4, Layer 2, and Layer 3. Ultimately, the span-morphing wing section achieved a span variation of 35.6%, which was consistent with the finite element results. In summary, the proposed span-morphing wing section based on MSHC structures exhibits not only a programmable nonlinear mechanical response but also good potential for engineering implementation.

The present prototype was designed as a laboratory-scale proof-of-concept demonstration using additively manufactured TPU/PLA components. Practical aerospace application will require further investigation of manufacturing scalability, aerospace-grade materials, integrated skin–structure design, and realistic loading conditions. In addition, aeroelastic effects, fatigue behavior, dynamic stability during repeated snap-through events, and local strain concentration in the curved beams should be systematically studied in future work.

## 5. Conclusions

A span-morphing wing section based on MSHC structures was proposed and investigated through structural design, numerical analysis, and functional experiments. Four curved-beam layers enabled a span variation of 35.6%. The MSHC structure provided both deformation and load-bearing capability, and the graded beam thickness realized the prescribed state-transition sequence during shortening and extension. Fluid–structure interaction analysis showed that lift increased with angle of attack and could be effectively regulated by span variation. Under the most severe loading condition considered, the maximum deformation of the flexible skin was only about 0.682% of the chord length, while the maximum stress in the span-morphing wing structure remained far below the material yield strength. Fabrication, assembly, and bidirectional morphing experiments demonstrated the engineering feasibility of the design.

It should be noted that the experimental prototype used uniform-thickness curved beams because of manufacturing limitations, while the programmed transition sequence in the numerical model was achieved using graded beam thicknesses. Therefore, the experiments demonstrate the bidirectional morphing function, but do not fully validate the programmed sequential morphing strategy, which will be further examined using higher-precision graded beams in future work. Overall, the proposed structure provides a lightweight and stable route for span-morphing wing design.

## Data Availability

The original contributions presented in this study are included in the article. Further inquiries can be directed to the corresponding author.

## Supplementary Materials

The following supporting information can be downloaded at https://www.mdpi.com/article/10.3390/ma19122678/s1. Video S1: Finite element simulation of the sequential morphing process of the span-morphing wing section.

## Abbreviations

The following abbreviations are used in this manuscript:

MSHC	Multistable honeycomb
ZPR	Zero Poisson ratio
NPR	Negative Poisson ratio
TPU	Thermoplastic polyurethane
PLA	Polylactic acid
